# Validation of a Novel Cuproptosis–Related Prognostic Gene Marker and Differential Expression Associated with Lung Adenocarcinoma

**DOI:** 10.3390/cimb45100536

**Published:** 2023-10-22

**Authors:** Tingting Liu, Jianshe Wei

**Affiliations:** Institute for Brain Sciences Research, School of Life Sciences, Henan University, Kaifeng 475004, China; ltt0808@henu.edu.cn

**Keywords:** cuproptosis, lung adenocarcinoma, overall survival, immune infiltration

## Abstract

Background: Cuproptosis induction is seen as a promising alternative for immunotherapies and targeted therapies in breast cancer. The objective of this research was to examine the prognostic and biological importance of cuproptosis-related genes (CRGs) in lung adenocarcinoma (LUAD). Methods: The following methods were used: GSE10072 dataset and TCGA database analysis, differential expression analysis of CRGs, and biological function (BP) and signaling pathway enrichment analysis, prognostic analysis and clinical analysis of CRGs, construction of the prognostic signature and RNA modified genes and miRNA analysis of CRGs in LUAD, immunoinfiltration analysis and immunohistochemical staining of DβH, UBE2D3, SOD1, UBE2D1 and LOXL2. Results: AOC1, ATOX1, CCL8, CCS, COX11, CP, LOXL2, MAP2K2, PDK1, SCO2, SOD1, UBE2D1, UBE2D3 and VEGFA showed significantly higher expression, while ATP7B, DβH, PDE3B, SLC31A2, UBE2D2, UBE2D4 and ULK2 showed lower expression in LUAD tissues than normal tissues. We also found that ATP7B (4%), AOC1 (3%) PDE3B (2%), DβH (2%), CP (1%), ULK2 (1%), PDK1 (1%), LOXL2 (1%) and UBE2D3 (1%) showed higher mutation frequencies. The univariate Cox analysis was used to identify CRGs that have prognostic value. It identified 21 genes that showed significant prognostic value, containing DβH, UBE2D3, SOD1, UBE2D1 and LOXL2. Patients with DβH up–expression have a longer survival time and patients with UBE2D3, SOD1, UBE2D1 and LOXL2 down–expression also have a longer survival time. hsa–miR–29c–3p, hsa–miR–29a–3p, hsa–miR–181c–5p, hsa–miR–1245a, etc., play an important role in the miRNA regulatory network, and in LUAD, miR–29a, miR–29c and miR–181c high expression survival was longer, and miR–1245a low expression survival was longer. We also performed an analysis to examine the relationships between DβH, LOXL2, SOD1, UBE2D1 and UBE2D3 and immune infiltration in LUAD, including B cells, CD8+ T cells, CD4+ T cells, macrophages, neutrophils and DCs. Conclusion: DβH, UBE2D3, SOD1, UBE2D1, and LOXL2 are potential candidates implicated in LUAD and can be further explored for their application as diagnostic, prognostic, and therapeutic biomarkers for LUAD.

## 1. Introduction

Lung cancer is one of the leading causes of cancer–related deaths and is classified into adenocarcinoma, squamous carcinoma, and large cell carcinoma (commonly referred as non–small cell lung cancer (NSCLC)) and small cell lung cancer based on pathological features [1]. Among the NSCLC subtypes, lung adenocarcinoma (LUAD) is the most common, accounting for 40–70% of cases [2]. Early diagnosis of NSCLC relies on lung biopsy through bronchoscopy, percutaneous needle biopsy, and exfoliative cytology [3]. Although liquid biopsy has emerged as a promising minimally invasive diagnostic method [4], the cost and limited availability of test equipment restrict its application. CT scan is the primary technique for monitoring lung cancer progression, but its limited resolution hinders the detection of minor advancements, including micro–metastasis and early drug resistance, let alone long–term disease outcome prediction. Therefore, there is an urgent need for a comprehensive evaluation approach in clinical practice [5].

The tumor microenvironment (TME) is a complex tissue environment consisting of various immune cells, stromal cells, and noncellular components [6]. As a result, there is a significant heterogeneity in the TME. Based on the levels of tumor–fighting effector cells and inflammatory cytokines, the TME can be classified into inflamed and non–inflamed types [7]. Additionally, several large–scale studies have shown the abundance of pre–existing infiltrated immune cells [8,9]. Cuproptosis, a newly defined concept of copper–dependent cell death, is associated with increased mitochondrial–dependent energy metabolism and accumulation of reactive oxygen species (ROS) [10]. Various studies have investigated the mechanisms of copper–induced cell death. Research on neurotoxicity suggests that copper disrupts the expression of host genes involved in olfactory signal transduction through the miRNA–mRNA pathway [11]. Interestingly, several studies have reported that cuproptosis–related genes (CRGs), such as antioxidant 1 copper chaperone (ATOX1), can impact cancer progression [12,13]. Importantly, Voli et al. discovered that variations in copper transporter 1 influence the expression of programmed cell death 1 ligand 1 (PD–L1), as well as the infiltration of CD8+ T cells and natural killer (NK) cells in the TME [14]. Copper pathways, including the ATOX–ATPase copper transporting Alpha (ATP7A)–lysyl oxidase (LOX) pathway, facilitate cancer cell metastasis, and inhibiting this pathway has been shown to impede breast cancer metastasis in vivo [15]. However, previous studies have mainly focused on one or two CRGs and their roles in cancer. A comprehensive analysis of multiple CRGs and their prognosis in LUAD is currently lacking. Therefore, we conducted a systematic correlation analysis of cuproptosis–related genes in LUAD for the first time.

In this study, we conducted data analysis on the GEO and TCGA databases to explore the expression of cuproptosis biomarkers in 58 LUAD samples and 49 healthy samples. We intended to comprehensively identify the molecular alterations and clinical relevance of CRGs in LUAD patients and investigated the impact of LUAD on immune cell infiltration through cuproptosis. Overall, our findings reveal a connection between this novel form of cell death and the malignancy of LUAD samples, suggesting the importance of further research on targeting this cellular mechanism.

## 2. Materials and Methods

### 2.1. Data Acquisition of LUAD Gene Expression Profile

The mRNA expression profile databases GSE10072 were downloaded from the GEO database (https://www.ncbi.nlm.nih.gov/, accessed on 23 March 2023) [16] in the data format MINIML. The dataset GSE10027 was located on the GPL96 Affymetrix Human Genome U133A Array. Moreover, with the help of the GEO2R analysis tool (http://www.ncbi.nlm.nih.gov/geo/geo2r, accessed on 23 March 2023) [17], we took the adjusted *p* < 0.05 as the cut–off criteria to analyze the differentially expressed genes between the LUAD and healthy samples.

### 2.2. Differential Expression of CRGs in LUAD

Initially, a total of 21 CRGs were obtained according to the previous report by Cai et al. [18]. Based on 21 CRGs expression profiles, we applied the unsupervised clustering analysis (“ConsensusClusterPlus” R package, version 2.60) classifying the 58 LUAD patients and 49 healthy patients into different clusters by using the k–means algorithm with 1000 iterations [19]. We used the dataset GSE10072 to map 21 CRG expression spectra and draw a boxplot. It was adopted to compare the expression of CRGs in various datasets using the R package “ggplot2”. For validation, we collected 21 CRGs expression values from the TCGA database. The digital focal–level copy number variation (CNV) values were computed from tumor samples using a “masked copy number fragment” file through GISTIC2 [20] at the item level, and subsequently filtered by a noise threshold of 0.3. To depict the frequency of CNV, a Cleveland dot plot was created using the R package “ggpubr”. The significance analysis for differences was conducted using unpaired Wilcoxon Rank Sum and Signed Rank Tests.

### 2.3. Gene Network and Enrichment Analysis of CRGs

The Gene Ontology (GO) and the Kyoto Encyclopedia of Genes and Genomes (KEGG) were used as references. Enrichment analysis was conducted using the R package “clusterProfiler” [21]. Heatmaps were plotted by https://www.bioinformatics.com.cn, accessed on 23 March 2023, an online platform for data analysis and visualization. We employed the Benjamini–Hochberg method for multiple correction, and a significance level of *p* < 0.05 was applied. The interaction of 21 CRGs was analyzed using the STRING database.

### 2.4. Prognostic Analysis and Clinical Staging of CRGs

In total, 21 CRGs were utilized in order to identify genes with univariate prognostic values through univariate Cox analysis [22]. Additionally, we calculated the 1–year survival, 3–year survival, and 5–year survival using the nearest neighbor estimation (NNE) method. In order to create a prognostic model for predicting overall survival (OS) in LUAD, we developed a nomogram based on the expression levels of DβH, UBE2D3, SOD1, UBE2D1, LOXL2, age, the T–, M–, N–stage, and clinical stage. R software (version 3.6.4) was employed to assess the expression differences of genes in LUAD across different clinical stage samples. We utilized the unpaired Student’s *t*–Test to determine the significance of differences between two pairs, and ANOVA was used to assess the variance among multiple groups of samples.

### 2.5. Construction of the Prognostic Signature of CRGs in LUAD

The risk score was calculated using the regression coefficients of the identified prognostic signature of CRGs for OS, respectively. Afterwards, patients were classified into high–risk and low–risk groups based on the median value of risk scores. The Kaplan–Meier survival curve was generated using the R package “ggsurvplot” to compare the OS between the high–risk and low–risk groups.

### 2.6. RNA Modified Genes and miRNA Analysis

The number of marker genes for the three types of RNA modifications (m1A, m5C, m6A) was determined. The GSCALite database, accessed on 24 March 2023 [23] was used to select the Cancer Genome Atlas Lung Adenocarcinoma (TCGA LUAD) and Genotype Tissue Expression Project (GTEx)–LUNG datasets as the background for analysis. The miRNA Network analysis function was chosen for analysis. The UALCAN database, accessed on 24 March 2023 [24] was used to assess the expression of miRNA and its correlation with survival in LUAD patients.

### 2.7. Analysis of Correlation with Immune Infiltration

A Tumor Immune Estimation Resource (TIMER; cistrome.shinyapps.io/timer, accessed on 25 March 2023) [25] was utilized to investigate the correlation between the expression of CRGs and the abundance of six immune cells (B cell, CD8+ T cell, CD4+ T cell, macrophages, neutrophils and dendritic cells (DCs)). Pearson correlation analysis was employed to assess the relationship between dopamine β–hydroxylase (DβH), lysyl oxidase like 2 (LOXL2), superoxide dismutase 1 (SOD1), ubiquitin conjugating enzyme E2 D1 (UBE2D1), UBE2D3 and somatic immunity in LUAD.

### 2.8. Single–Cell–Type Analysis and Immunohistochemical Staining

The analysis of gene clustering for single–cell types was conducted using the Human Protein Atlas (HPA) database (https://www.proteinatlas.org/, accessed on 26 March 2023) [26]. In order to confirm the differential expression of DβH, UBE2D1, UBE2D3, SOD1, and LOXL2 between normal and tumor tissues, immunohistochemical staining images of normal breast tissues and breast cancer tissues were obtained from the HPA.

## 3. Results

### 3.1. Data Acquisition of LUAD Genes Expression Profile

**Figure 1:**
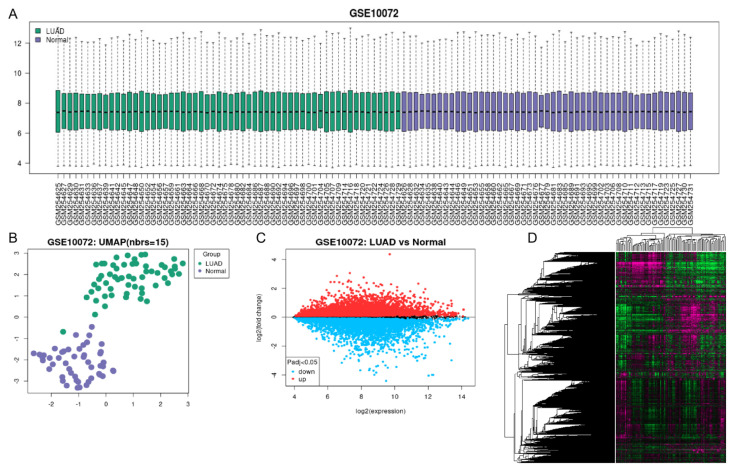
Gene chip data information ((**A**): the gene chip expression profiles of 107 lung tissue samples, including 58 LUAD patients and 49 healthy patients; (**B**): PCA analysis; (**C**): differential gene volcano map; (**D**): differential gene clustering, compared with healthy individuals, pink represented high expression genes and green represented low expression genes.).

The GSE10072 dataset was analyzed using the GEO database. This dataset includes the gene chip expression profiles of 107 lung tissue samples, consisting of 58 LUAD patients and 49 healthy individuals (Figure 1A). The samples exhibit a high degree of correlation (Figure 1B). In total, 11,916 genes were identified with significant differences (*p* < 0.05), including 6555 up–regulated genes and 5361 down–regulated genes (Figure 1C). The clustering analysis results are displayed in Figure 1D, with pink representing up–regulated genes and green representing down–regulated genes.

### 3.2. Differential Expression of CRGs in LUAD

**Figure 2:**
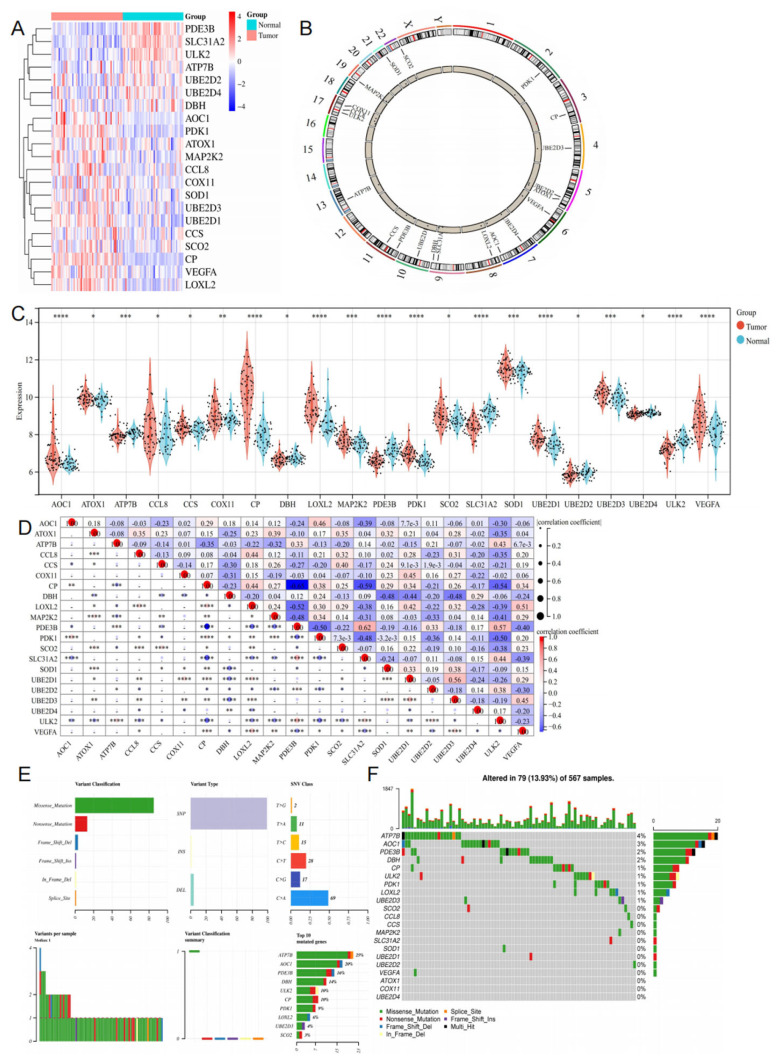
Expression and genetic alteration of CRGs in LUAD ((**A**): the expression patterns of 21 CRGs are presented in the heatmap; (**B**): the location of 21 CRGs on chromosomes; (**C**): boxplots showed the expression of 21 CRGs between LUAD and healthy groups; (**D**): correlations between the expression of cuproptosis regulators; (**E**,**F**): the CNV and mutation frequency and classification of 21 CRGs in LUAD. * *p* < 0.05, ** *p* < 0.01, *** *p* < 0.001, **** *p* < 0.0001.).

We curated a catalog of 21 genes (amine oxidase copper containing 1 (AOC1), ATOX1, ATP7B, CCL8, copper chaperone superoxide (CCS), cytochrome C oxidase copper chaperone (COX11), ceruloplasmin (CP), DβH, LOXL2, mitogen–activated protein kinase 2 kinase 2 (MAPK2K2), phosphodiesterase 3B (PDE3B), pyruvate dehydrogenase kinase 1 (PDK1), synthesis of cytochrome C oxidase 2 (SCO2), solute carrier family 31 member 2 (SLC31A2), SOD1, UBE2D1, UBE2D2, UBE2D3, UBE2D4, unc–51 like autophagy activating kinase 2 (ULK2), vascular endothelial growth factor A (VEGFA)) that function closely with cuproptosis. In the comparison of differentially expressed genes between tumor and normal tissues in LUAD patients from the GSE10072 dataset and TCGA, the genes AOC1, ATOX1, CCL8, CCS, COX11, CP, LOXL2, MAP2K2, PDK1, SCO2, SOD1, UBE2D1, UBE2D3, and VEGFA showed significantly higher expression in LUAD tissues while ATP7B, DβH, PDE3B, SLC31A2, UBE2D2, UBE2D4, and ULK2 showed lower expression compared to normal tissues (Figure 2A–C, Supplementary Table S1). Additionally, we investigated the correlation between the expression of different genes, which revealed strong associations (Figure 2D). Missense mutation was the most frequent classification of mutations (Figure 2E). The most prevalent variant type was single–nucleotide polymorphism (SNP), with C > A (69) being the most common single–nucleotide variant (SNV). We also observed higher mutation frequencies in ATP7B (4%), AOC1 (3%), PDE3B (2%), DβH (2%), CP (1%), ULK2 (1%), PDK1 (1%), LOXL2 (1%), and UBE2D3 (1%) compared to others (Figure 2F).

### 3.3. Functional Enrichment and Protein–Protein Interaction Analysis of CRGs

**Figure 3:**
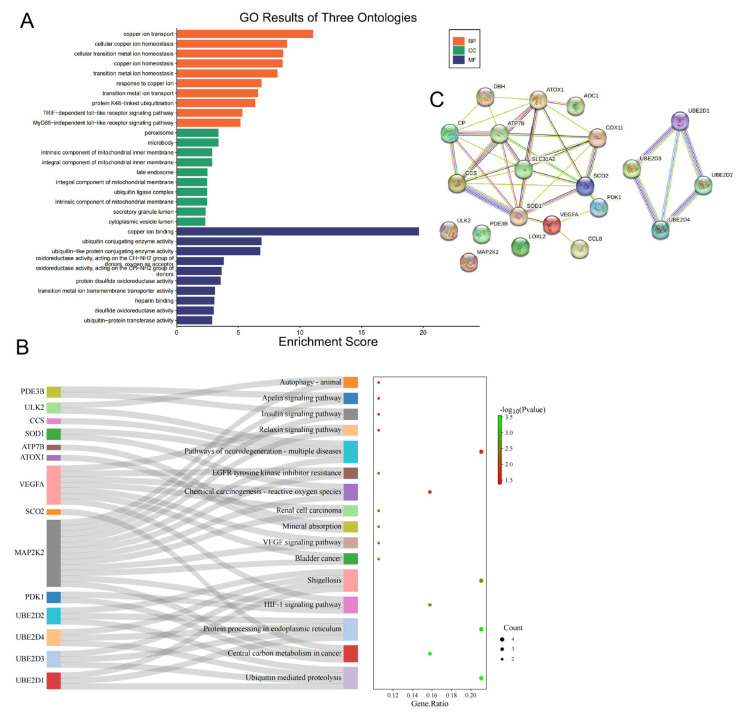
GO, pathway enrichment and PPI analysis of CRGs in LUAD patients ((**A**): the enriched item in the gene ontology analysis; (**B**): the enriched item in the Kyoto Encyclopedia of Genes and Genomes analysis. The size of circles represents the number of enriched genes. (**C**): PPI of CRGs. BP: biological process, CC: cellular component, MF: molecular function and CRG: cuproptosis–related gene, PPI: protein–protein interaction).

To understand the biological functions of CRGs, we analyzed the relevant pathways using the GO and KEGG databases. The biological processes mainly involved in the GO analysis were related to copper ion transport, cellular copper ion homeostasis, cellular transition metal ion homeostasis, copper ion homeostasis, transition metal ion homeostasis, response to copper ions, and transition metal ion transport. The cellular components mainly involved in the GO analysis were peroxisome, microbody, late endosome, ubiquitin ligase complex, secretory granule lumen, cytoplasmic vesicle lumen, vesicle lumen, and mitochondrial matrix. The molecular functions mainly involved in the GO analysis were copper ion binding, ubiquitin conjugating enzyme activity, and ubiquitin–like protein conjugating enzyme activity (Figure 3A). In addition, the enrichment analysis of the KEGG pathway showed that the 21 CRGs were mostly associated with ubiquitin–mediated proteolysis, protein processing in the endoplasmic reticulum, shigellosis, pathways of neurodegeneration–multiple diseases, central carbon metabolism in cancer, the HIF–1 signaling pathway, chemical carcinogenesis–reactive oxygen species, and the VEGF signaling pathway (Figure 3B). A protein–protein interaction (PPI) analysis was conducted to explore the interactions of the CRGs. The analysis revealed 21 nodes, 40 edges, an average node degree of 3.81, an average local clustering coefficient of 0.611, and a PPI enrichment *p*–value < 1.0 × 10^−16^ (Figure 3C).

#### Prognostic Analysis and Clinical Staging of CRGs

**Figure 4:**
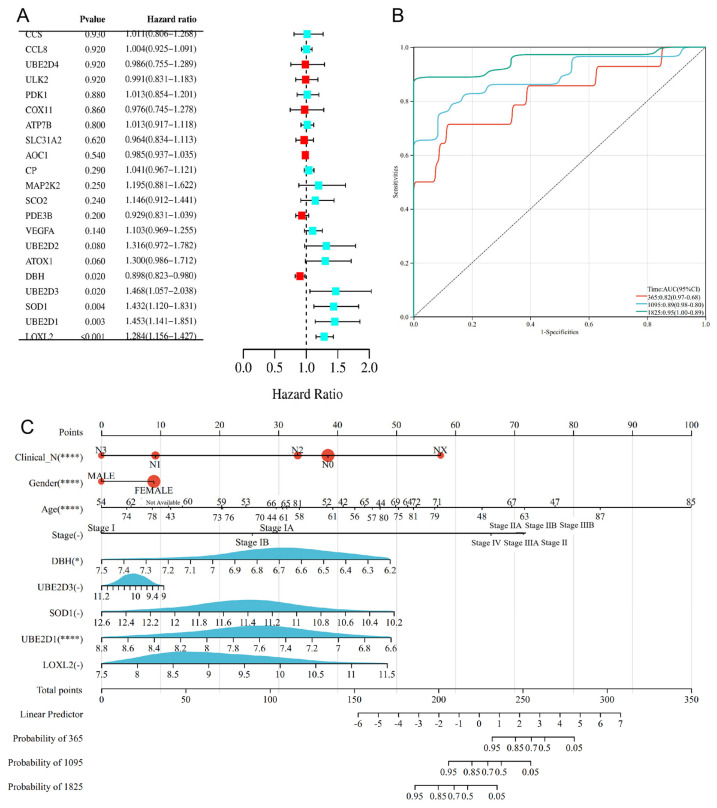
Prognostic analysis and clinical staging of CRGs ((**A**): prognostic analysis of cuproptosis–related genes using univariate Cox regression; (**B**): ROCs for one–year, three–year and five–year survival prediction; (**C**): nomogram for OS prediction, with N–stage, gender, age and clinical stage, and the expression level of DβH, UBE2D3, SOD1, UBE2D1, LOXL2 applied as parameters. * *p* < 0.05, **** *p* < 0.0001).

The univariate Cox analysis was used to select CRGs with prognostic value and identified 21 genes with significant prognostic potential, containing DβH (HR = 0.898 (0.823–0.980), *p* = 0.020), UBE2D3 (HR = 1.468 (1.057–2.038), *p* = 0.020), SOD1 (HR = 1.432 (1.120–1.831), *p* = 0.004), UBE2D1 (HR = 1.453 (1.141–1.851), *p* = 0.003) and LOXL2 (HR = 1.284 (1.156–1.427), *p* < 0.001) (Figure 4A). The prediction accuracy evaluated by AUCs was reported to be 0.82, 0.89 and 0.95 in the 1–year, 3–year and 5–year ROC curves, respectively (Figure 4B). The expression level of DβH, UBE2D3, SOD1, UBE2D1 and LOXL2, the N–stage, gender, age and the clinical stage were eventually applied as parameters (Figure 4C). The clinical staging (T–, N–, M–stage) of CRGs is shown in Supplementary Table S2. *p* < 0.05 demonstrates a statistically significant difference.

### 3.4. Construction of the Prognostic Signature of CRGs in LUAD

**Figure 5:**
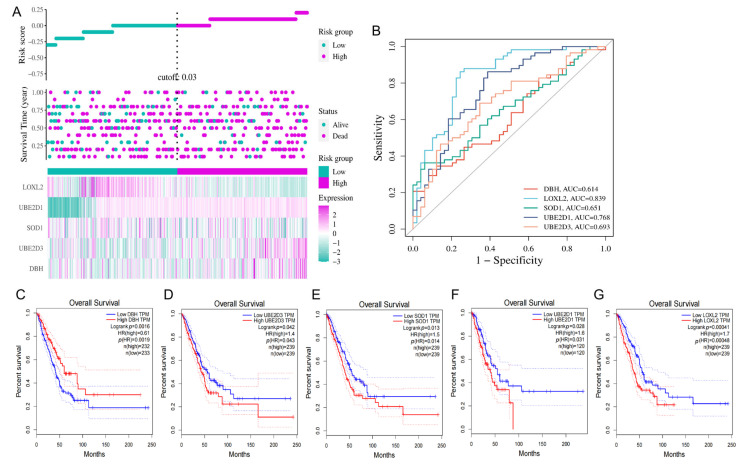
Clinical relevance of CRGs in the LUAD patients of TCGA ((**A**): the distribution of risk score, survival status and the expression of prognostic CRGs; (**B**): ROCs for DβH, LOXL2, SOD1, UBE2D1 and UBE2D3 survival prediction; Kaplan–Meier plot for the expression of (**C**) DβH (**D**) UBE2D3 (**E**) SOD1 (**F**) UBE2D1 and (**G**) LOXL2 and overall survival).

For overall survival outcomes of LUAD patients, LOXL2, UBE2D1, SOD1, UBE2D3, and DβH genes were selected to create a prognostic score based on their regression coefficients (Figure 5A). We used a weighted risk score incorporating all these genes to estimate the 0.25–, 0.5–, 0.75– and 1–year overall survival rates. The accuracy of prediction, as measured by the AUC (Area Under the Curve), was reported to be 0.614, 0.839, 0.651, 0.768, and 0.693 in the DβH, LOXL2, SOD1, UBE2D1, and UBE2D3 ROC curves, respectively (Figure 5B). Patients with higher expression levels of the DβH gene have a longer survival time, while those with lower expression levels of UBE2D3, SOD1, UBE2D1, and LOXL2 genes have a longer survival time (Figure 5C–G).

### 3.5. RNA Modified Genes and miRNA Analysis

**Figure 6:**
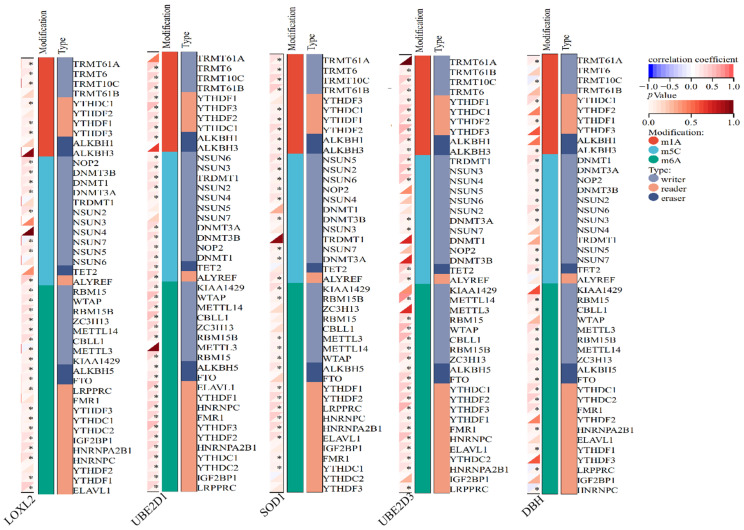
RNA modified gene analysis, relationship between DβH, UBE2D3, SOD1, UBE2D1 and LOXL2 on RNA modified genes in LUAD. Compared with healthy individuals, * *p* < 0.05.

**Figure 7:**
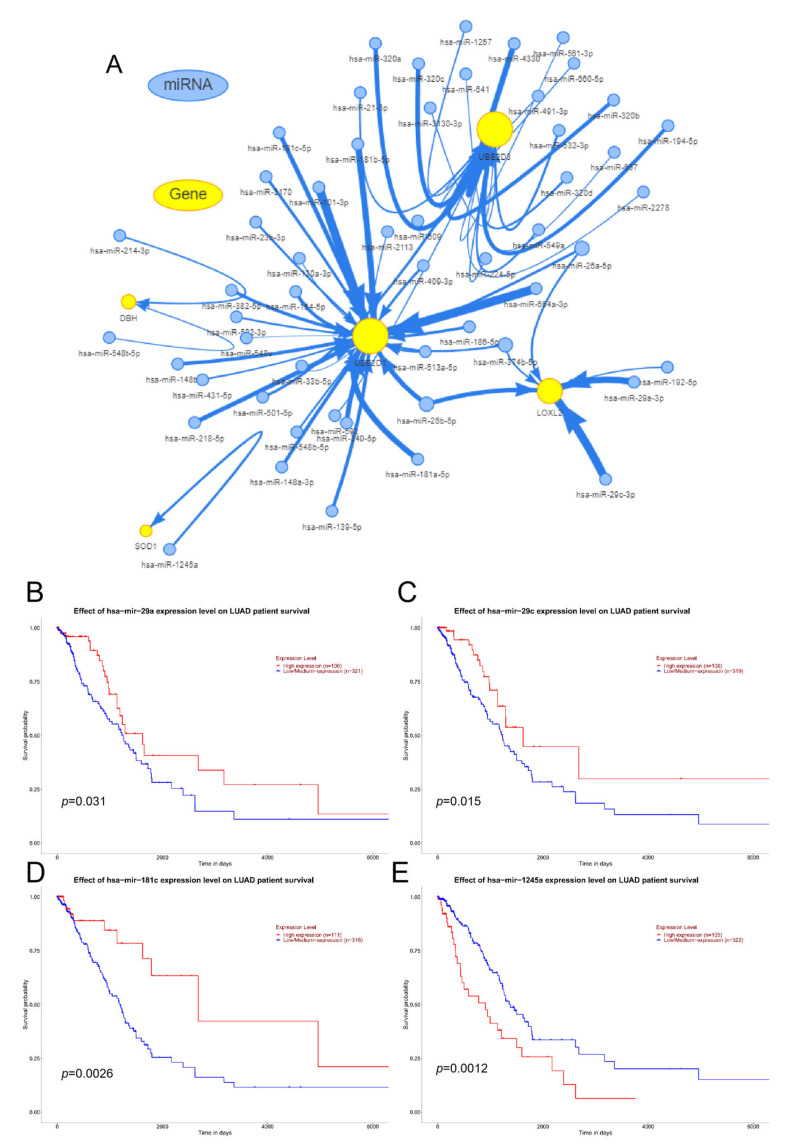
miRNA analysis ((**A**): DβH, UBE2D3, SOD1, UBE2D1 and LOXL2 and miRNA regulatory networks; UALCAN for the expression of (**B**) miR–29a, (**C**) miR–29c, (**D**) miR–181c and (**E**) miR–1245a and overall survival).

Epigenetic modifications, such as DNA methylation, RNA modification, and histone modifications, have been widely reported to play an important role in lung cancer development and in other pulmonary diseases. Similar to epigenetic modifications in DNA and histones, these RNA marks can be deposited, erased, and recognized by a set of specialized protein machineries, namely writers, erasers, and readers. While N6–methyladenosine (m6A) is the most common internal modification in mRNA, there are several other important modifications present in both mRNA and non–coding RNA (ncRNA), including N1–methyladenosine (m1A), 5–methylcytosine (m5C), 7–methylguanosine (m7G), pseudouridine (Ψ, psi), and adenosine–to–inosine (A–to–I) conversion [27]. TRMT61A (m1A) and KIAA1429 (m6A) [28] regulated the RNA writer, YTHDF1 (m1A and m6A) [29] and YLAREF (m5C) regulated the RNA reader, ALKBH1 (m1A) and FTO (m6A) [30] regulated the RNA eraser, and the expression of DβH, UBE2D3, SOD1, UBE2D1 and LOXL2 was positively correlated with LUAD (Figure 6). The miRNA expression of genes was shown in Figure 7A, and hsa–miR–29c–3p, hsa–miR–29a–3p, hsa–miR–181c–5p, hsa–miR–1245a, etc., play an important role in the miRNA regulatory network. Among them, in LUAD, miR–29a (*p* = 0.031), miR–29c (*p* = 0.015) and miR–181c (*p* = 0.0026) high expression survival was longer, and miR–1245a (*p* = 0.0012) low expression survival was longer (Figure 7B–E).

### 3.6. Correlation between the Expression of CRGs and Levels of Immune Infiltration in LUAD

**Figure 8:**
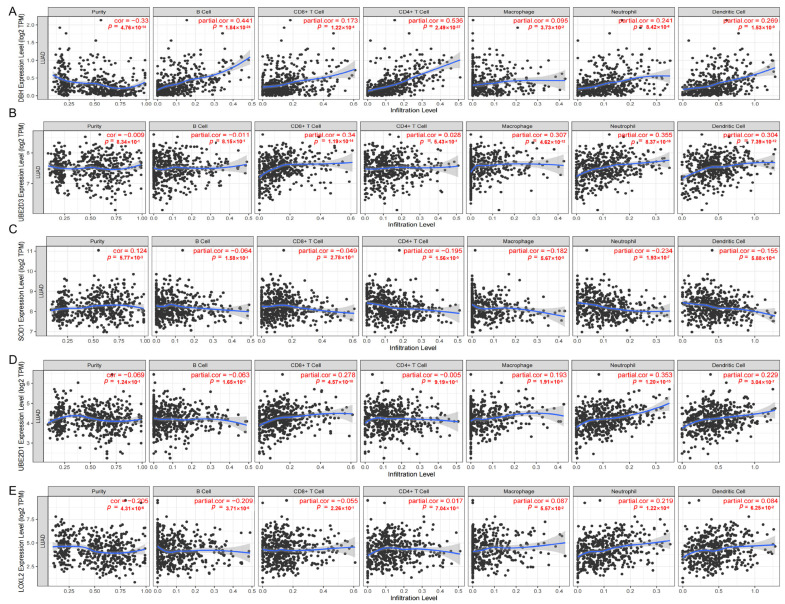
The correlation between (**A**) DβH, (**B**) UBE2D3, (**C**) SOD1, (**D**) UBE2D1, and (**E**) LOXL2 expression and immune infiltration in LUAD in the TIMER database.

It is unknown whether CRGs have an impact on the recruitment of immune cells in the tumor microenvironment and subsequently affect the prognosis of LUAD. For LUAD, the infiltration of immune cells is often closely related to tumor development and prognosis. Studies have shown that the infiltration of immune cells can play a dual role: on the one hand, they can recognize and kill tumor cells, thus inhibiting the growth and spread of tumors; on the other hand, tumor cells can also evade the attack of the immune system by interacting with immune cells and promote the progression of tumors [31,32]. In order to address this, we conducted an analysis to examine the associations between the expression levels of DβH, LOXL2, SOD1, UBE2D1, UBE2D3 and somatic immunity in LUAD. Our findings revealed that the expression levels of DβH, LOXL2, and UBE2D1 were positively correlated with somatic immunity, whereas the expression level of SOD1 showed a negative correlation with somatic immunity (Supplementary Figure S1). We also performed an analysis to examine the relationships between DβH, LOXL2, SOD1, UBE2D1 and UBE2D3 and immune infiltration in LUAD, including B cells, CD8+ T cells, CD4+ T cells, macrophages, neutrophils and DCs. The expression level of DβH was positively associated with immune infiltration (Figure 8A). The expression level of UBE2D3 was positively associated with the immune infiltration level of CD8+ T cells, macrophages, neutrophils and DCs and negatively correlated with B cells (Figure 8B). The expression level of SOD1 was negatively associated with the immune infiltration level of CD4+ T cells, macrophages, neutrophils and DCs (Figure 8C). The expression level of UBE2D1 was positively associated with the immune infiltration level of CD8+ T cells, macrophages, neutrophils and DCs (Figure 8D). The expression level of LOXL2 was positively associated with the immune infiltration level of neutrophils and negatively correlated with B cells (Figure 8E).

### 3.7. Single–Cell–Type Analysis and Immunohistochemical Staining

**Figure 9:**
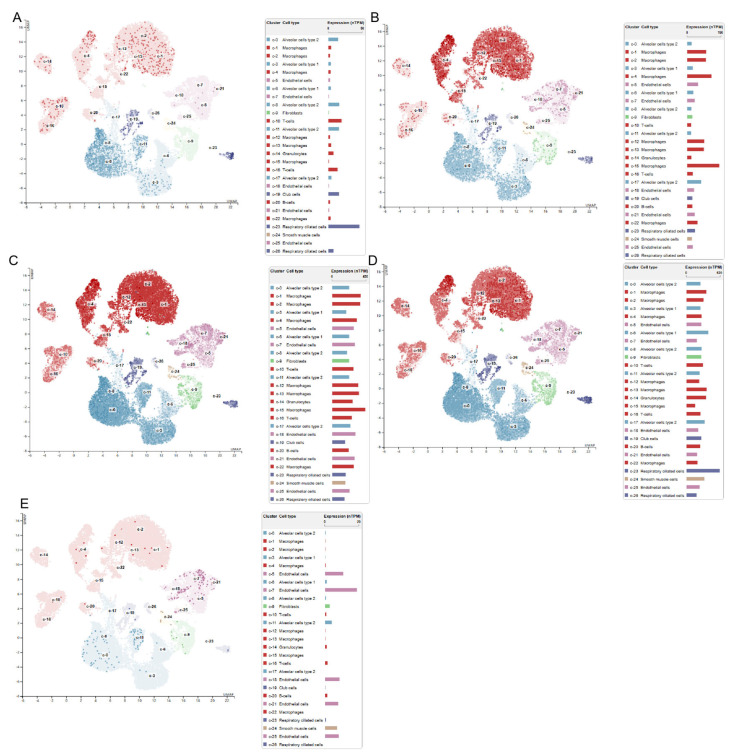
Single–cell–type analysis, including (**A**) DβH, (**B**) UBE2D1, (**C**) UBE2D3, (**D**) SOD1, and (**E**) LOXL2.

Through the clustering analysis of gene cell types, they were mainly distributed in c–0 (macrophages), c–1 (alveolar cells type 2), c–2 (macrophages), c–3 (T–cells), c–4 (granulocytes), c–5 (fibroblasts), c–6 (alveolar cells, type 2), c–7 (Club cells), c–8 (respiratory ciliated cells), c–9 (endothelial cells), c–10 (alveolar cells, type 1). UBE2D1 was mainly expressed in macrophages, endothelial cells and T–cells. UBE2D3 was mainly expressed in all the above cell types. SOD1 was mainly expressed in respiratory ciliated cells, macrophages and fibroblasts. LOXL2 was mainly expressed in endothelial cells and fibroblasts (Figure 9A–E). The immunohistochemical staining images of normal lung tissues and tumor tissues were acquired from the HPA database. The images revealed a significantly higher expression level of DβH, UBE2D1, UBE2D3, SOD1, and LOXL2 in the lung tumor tissue compared to the normal tissue. The protein expression was predominantly observed in the cytoplasm and nucleus (Supplementary Figure S2A–E).

## 4. Discussion

Copper is an essential element for cell proliferation and cell death. Additionally, it serves as a necessary cofactor for enzymes and transporters [33]. Dysregulation of copper metabolism can lead to cytotoxic effects and oxidative stress responses in various cell types [34,35]. We define copper–dependent cell proliferation as cuproplasia. On the other hand, copper–dependent cell death is referred to as cuproptosis, which is likely mediated by increased energy metabolism in mitochondria and accumulation of reactive oxygen species (ROS) [36]. Importantly, cuproptosis has been found to be closely associated with tumorigenesis, progression, and metastasis. Li et al. demonstrated that the IL–17–STEAP4–XIAP axis, activated by cuproptosis, can transform colon inflammation into cancer [37]. Petris et al. revealed that silencing ATP7A can inhibit the progression and metastasis of lung cancer by altering the activity of LOX family enzymes [15]. Moreover, several studies have uncovered a strong correlation between cuproptosis and immune cell infiltration. Paredes et al. reported that mutation of MAP2K1 can influence the abundance of macrophages, mature dendritic cells, regulatory T cells, and cytotoxic lymphocytes [38]. Tan et al. showed that ceruloplasmin plays a crucial role in immune infiltration in breast cancer [39].

Tumor (T) refers to the tumor factor, that is, including the size of the tumor, the depth of tumor invasion, the corresponding invasion or adjacency of the tumor with surrounding tissues, organs, blood vessels, or nerves, thereby determining the T stage. However, different organs are specific, and the T stage can be different, such as lung tumors are generally divided according to their maximum diameter, such as T1: 3 cm, T2: 3–5 cm, T3: 5–7 cm, and T4 > 7 cm. In addition, digestive system tumors are mainly analyzed by the depth of invasion, so different tumors have certain particularities; N: refers to the stage of lymph nodes, depending on whether there is lymph node metastasis, if there is no lymph node metastasis, it is N0. If there is lymph node metastasis, it is necessary to determine whether it is local lymph node metastasis, distant lymph node metastasis, and the number of specific lymph node metastases, so as to determine whether it is N1, N2, or N3. But the later the stage, the worse the corresponding prognosis; M: refers to whether there is distant metastasis; if no distant metastasis occurs through the blood system, it is M0. If distant metastases occur, it is M1, which is stage IV and relatively advanced disease [40,41]. Our findings revealed that, compared with healthy humans, the AOC1, CCS, DβH, UBE2D1, UBE2D2 and ULK2 in LUAD patients were statistically different at the T–stage, the ATOX1, ATP7B, CP, PDE3B, UBE2D3 and UBE2D4 in LUAD patients were statistically different at the N–stage, the LOXL2 in LUAD patients was statistically different at the T–, N–stage; the SOD1 in LUAD patients was statistically different at the M–stage.

We found that the patients with DβH up–expression have a longer survival time, while patients with UBE2D3, SOD1, UBE2D1 and LOXL2 down–expression have a longer survival time. The DβH, LOXL2, UBE2D1 expression level was positively correlated with somatic immunity and the SOD1 expression level was negatively correlated with somatic immunity. The protein encoded by DβH is an oxidoreductase belonging to the copper type II, ascorbate–dependent monooxygenase family. The encoded protein, expressed in neuroscretory vesicles and chromaffin granules of the adrenal medulla, catalyzes the conversion of dopamine to norepinephrine, which functions as both a hormone and as the main neurotransmitter of the sympathetic nervous system. The enzyme encoded by this gene exists in both soluble and membrane–bound forms, depending on the absence or presence, respectively, of a signal peptide. Mutations in this gene cause dopamine beta–hydroxylate deficiency in human patients, characterized by deficits in autonomic and cardiovascular function, including hypotension and ptosis. Polymorphisms in this gene may play a role in a variety of psychiatric disorders [42]. DβH is involved in the synthesis and metabolism of dopamine, a neurotransmitter involved in many physiological processes, including regulation of mood, motor control, and cognitive function. Some studies have suggested that abnormal levels of dopamine may be associated with the onset and development of certain cancer types. For example, tumors such as breast and prostate cancer can influence their growth and metastasis through pathways related to dopamine secretion and metabolism. In addition, several studies have also found that genetic variants associated with dopamine are associated with an increased risk of certain cancers [43,44]. DβH is highly expressed in most cancers, including melanoma and pheochromocytoma, while DβH is minimally expressed in LUAD in our study. Therefore, we speculate that the role of dopamine in the lung is to affect the pulmonary blood flow by regulating the contraction and relaxation of pulmonary blood vessels. It dilates the blood vessels in the lungs and increases blood flow, thereby improving the exchange of oxygen and carbon dioxide. In addition, dopamine can also promote bronchiectasis, reduce bronchospasm, and help improve breathing, thus improving the clinical symptoms of LUAD patients. Therefore, DβH is expected to be a novel marker for the treatment of LUAD. The modification of proteins with ubiquitin is an important cellular mechanism for targeting abnormal or short–lived proteins for degradation. Ubiquitination involves at least three classes of enzymes: ubiquitin–activating enzymes, or E1s, ubiquitin–conjugating enzymes, or E2s, and ubiquitin–protein ligases, or E3s. This gene encodes a member of the E2 ubiquitin–conjugating enzyme family. UBE2D1 and UBE2D3 enzymes function in the ubiquitination of the tumor–suppressor protein p53, which are induced by an E3 ubiquitin–protein ligase [45]. Ubiquitin–modified proteins are closely related to the regulation of the cell cycle, DNA repair, apoptosis and other biological processes. Overexpression of UBE2D1 and UBE2D3 is common in LUAD tissues and is related to the malignancy and prognosis of LUAD. High expression of UBE2D1 and UBE2D3 can promote the proliferation, invasion and metastasis of tumor cells, and inhibit the apoptosis of tumor cells [46]. Therefore, they may be a potential therapeutic target for LUAD. SOD1 in lung cancer cells promotes growth without affecting cell cycle progression. Instead, cells overexpressing SOD1 had a lower frequency of basal apoptosis, implying that SOD1 promotes growth by increasing survival [47]. The authors speculated that, when SOD1 activity is low, there is an abundant supply of ROS that prevents the oxidation of tyrosine phosphates [48]. This causes the down–regulation of growth–promoting signals from tyrosine kinase receptors and this may be the mechanism by which ROS impair growth. Given our observation that overexpression of SOD1 leads to increased growth, it seems likely that cells with high levels of SOD1 have a selective advantage during tumorigenesis. Thus, SOD1 might be a valuable therapeutic target. LOXL2 is a member of the amine oxidase family, which plays a role in the formation of crosslinks in stromal collagens and elastin, as well as cell motility, tumor development, and progression. The expression of LOXL2 in tumor tissues and cancer cell lines has been found to be both down–regulated and up–regulated, suggesting differing roles in cancer. In a study by Zhan et al., LOXL2 mRNA expression was significantly down–regulated in NSCLC, particularly in patients with LUAD [49]. LOXL2 is involved in multiple biological processes of LUAD, including cell proliferation, invasion and metastasis. It can affect the migration and invasion ability of tumor cells by regulating the structure and stiffness of extracellular matrix [50]. In addition, LOXL2 may also promote the development of LUAD by influencing cell–cell interactions in the tumor microenvironment [51]. Therefore, LOXL2 may be an important therapeutic target for LUAD. This down–regulation correlated with poorer differentiation, a higher N–stage, and advanced pathologic T–, N–, and M–stages. We also analyzed the relationship between LOXL2 and immune infiltration in LUAD, including B cells, CD8+ T cells, CD4+ T cells, macrophages, neutrophils, and dendritic cells (DCs). Changes in intracellular copper levels may affect the tumor immune microenvironment and are associated with cancer progression. Previous studies have identified some genes related to cuproptosis, but the molecular subtypes of cuproptosis in LUAD have not been thoroughly studied. These genes could be used as potential biomarkers to help diagnose LUAD. By testing the patient’s genome, it is possible to determine if abnormal changes in these genes are present. For example, mutations in the SOD1 gene are associated with oxidative stress, and mutations in the LOXL2 gene are associated with tumor proliferation and invasion. By detecting changes in these genes, it can help doctors make an early diagnosis and carry out precise treatment. The expression level of these genes may be related to the prognosis of LUAD patients. As shown in this study, high expression of UBE2D3, SOD1, UBE2D1 and LOXL2 is associated with malignancy and poor prognosis of LUAD. By monitoring the expression levels of these genes, patient prognosis can be assessed and individualized treatment options can be recommended.

This study presented a comprehensive overview of cuproptosis and copper metabolism–related cell death genes in LUAD. The expression levels of LOXL2, UBE2D1, UBE2D3 were significantly higher, while DβH showed lower expression in LUAD tissues compared to normal tissues. These cell death genes also have the potential to influence LUAD survival and interact with other pathways. The impact of these genes on the cancer microenvironment suggests their potential value in cancer therapies. Therefore, LOXL2, UBE2D1, UBE2D3, and DβH can be considered as potential candidates for cancer diagnosis, prognosis, and therapeutic biomarkers. Nevertheless, this study has some limitations, and further investigation is required to elucidate the underlying mechanism of cuproptosis in LUAD both in vitro and in vivo.

## Data Availability

All data and material generated or analyzed during this study are included in this published article.

## Supplementary Materials

The following supporting information can be downloaded at: https://www.mdpi.com/article/10.3390/cimb45100536/s1, Figure S1: Somatic immunity score between (A) DβH, (B) UBE2D3, (C) SOD1, (D) UBE2D1, and (E) LOXL2 expression; Figure S2: Immunohistochemical staining and immunofluorometric assay, including (A) DβH, (B) UBE2D1, (C) UBE2D3, (D) SOD1, and (E) LOXL2; Table S1: Results of differential expression of CRGs in GSE10072 and TCGA datasets; Table S2: The clinical staging of CRGs.

## References

[1] Ruiz-Cordero R., Devine W.P. (2020). Targeted Therapy and Checkpoint Immunotherapy in Lung Cancer. Surg. Pathol. Clin..

[2] Kadara H., Choi M., Zhang J., Parra E., Rodriguez-Canales J., Gaffney S., Zhao Z., Behrens C., Fujimoto J., Chow C. (2018). Whole-exome sequencing and immune profiling of early-stage lung adenocarcinoma with fully annotated clinical follow-up. Ann. Oncol..

[3] Postmus P.E., Kerr K.M., Oudkerk M., Senan S., Waller D.A., Vansteenkiste J., Escriu C., Peters S., ESMO Guidelines Committee (2017). Early and Locally Advanced Non-Small-Cell Lung Cancer (NSCLC): ESMO Clinical Practice Guidelines for Diagnosis, Treatment and Follow-up. Ann. Oncol..

[4] Li W., Liu J.-B., Hou L.-K., Yu F., Zhang J., Wu W., Tang X.-M., Sun F., Lu H.-M., Deng J. (2002). Liquid biopsy in lung cancer: Significance in diagnostics, prediction, and treatment monitoring. Mol. Cancer.

[5] Duan Q., Zhang H., Zheng J., Zhang L. (2020). Turning Cold into Hot: Firing up the Tumor Microenvironment. Trends Cancer.

[6] Alonso R.C., Hidalgo L.H., Moreno E., Correa C.P., de Vega V.M. (2012). Papel de las técnicas de imagen en la nueva clasificación TNM del carcinoma broncogénico no microcítico [Role of imaging techniques in the TNM classification of non-small cell broncho-genic carcinoma]. Radiologia.

[7] Gajewski T.F. (2015). The Next Hurdle in Cancer Immunotherapy: Overcoming the Non–T-Cell–Inflamed Tumor Microenvironment. Semin. Oncol..

[8] Havel J.J., Chowell D., Chan T.A. (2019). The evolving landscape of biomarkers for checkpoint inhibitor immunotherapy. Nat. Rev. Cancer.

[9] Tumeh P.C., Harview C.L., Yearley J.H., Shintaku I.P., Taylor E.J.M., Robert L., Chmielowski B., Spasic M., Henry G., Ciobanu V. (2014). PD-1 blockade induces responses by inhibiting adaptive immune resistance. Nature.

[10] Ge E.J., Bush A.I., Casini A., Cobine P.A., Cross J.R., DeNicola G.M., Dou Q.P., Franz K.J., Gohil V.M., Gupta S. (2022). Connecting copper and cancer: From transition metal signalling to metalloplasia. Nat. Rev. Cancer.

[11] Wang L., Bammler T.K., Beyer R.P., Gallagher E.P. (2013). Copper-Induced Deregulation of microRNA Expression in the Zebrafish Olfactory System. Environ. Sci. Technol..

[12] Blockhuys S., Wittung-Stafshede P. (2017). Roles of Copper-Binding Proteins in Breast Cancer. Int. J. Mol. Sci..

[13] Kardos J., Héja L., Simons Á., Jablonkai I., Kovács R., Jemnitz K. (2018). Copper signalling: Causes and consequences. Cell Commun. Signal..

[14] Voli F., Valli E., Lerra L., Kimpton K., Saletta F., Giorgi F.M., Mercatelli D., Rouaen J.R.C., Shen S., Murray J.E. (2020). Intratumoral Copper Modulates PD-L1 Expression and Influences Tumor Immune Evasion. Cancer Res..

[15] Shanbhag V., Jasmer-McDonald K., Zhu S., Martin A.L., Gudekar N., Khan A., Ladomersky E., Singh K., Weisman G.A., Petris M.J. (2019). ATP7A delivers copper to the lysyl oxidase family of enzymes and promotes tumorigenesis and metastasis. Proc. Natl. Acad. Sci. USA.

[16] Clough E., Barrett T. (2016). The Gene Expression Omnibus Database. Methods Mol. Biol..

[17] Barrett T., Wilhite S.E., Ledoux P., Evangelista C., Kim I.F., Tomashevsky M., Marshall K.A., Phillippy K.H., Sherman P.M., Holko M. (2013). NCBI GEO: Archive for functional genomics data sets—Update. Nucleic Acids Res..

[18] Cai Z., He Y., Yu Z., Hu J., Xiao Z., Zu X., Li Z., Li H. (2022). Cuproptosis-related modification patterns depict the tumor microenvironment, precision immunotherapy, and prognosis of kidney renal clear cell carcinoma. Front. Immunol..

[19] Wilkerson M.D., Hayes D.N. (2010). ConsensusClusterPlus: A class discovery tool with confidence assessments and item tracking. Bioinformatics.

[20] Mermel C.H., Schumacher S.E., Hill B., Meyerson M.L., Beroukhim R., Getz G. (2011). GISTIC2.0 facilitates sensitive and confident localization of the targets of focal somatic copy-number alteration in human cancers. Genome Biol..

[21] Yu G., Wang L.-G., Han Y., He Q.-Y. (2012). clusterProfiler: An R Package for Comparing Biological Themes Among Gene Clusters. OMICS.

[22] Heagerty P.J., Zheng Y. (2005). Survival Model Predictive Accuracy and ROC Curves. Biometrics.

[23] Liu C.-J., Hu F.-F., Xia M.-X., Han L., Zhang Q., Guo A.-Y. (2018). GSCALite: A web server for gene set cancer analysis. Bioinformatics.

[24] Chandrashekar D.S., Bashel B., Balasubramanya S.A.H., Creighton C.J., Ponce-Rodriguez I., Chakravarthi B.V.S.K., Varambally S. (2017). UALCAN: A portal for facilitating tumor subgroup gene expression and survival analyses. Neoplasia.

[25] Li T., Fan J., Wang B., Traugh N., Chen Q., Liu J.S., Li B., Liu X.S. (2017). TIMER: A Web Server for Comprehensive Analysis of Tumor-Infiltrating Immune Cells. Cancer Res..

[26] Sjöstedt E., Zhong W., Fagerberg L., Karlsson M., Mitsios N., Adori C., Oksvold P., Edfors F., Limiszewska A., Hikmet F. (2020). An atlas of the protein-coding genes in the human, pig, and mouse brain. Science.

[27] Teng P.-C., Liang Y., Yarmishyn A.A., Hsiao Y.-J., Lin T.-Y., Lin T.-W., Teng Y.-C., Yang Y.-P., Wang M.-L., Chien C.-S. (2021). RNA Modifications and Epigenetics in Modulation of Lung Cancer and Pulmonary Diseases. Int. J. Mol. Sci..

[28] Schwartz S., Mumbach M.R., Jovanovic M., Wang T., Maciag K., Bushkin G.G., Mertins P., Ter-Ovanesyan D., Habib N., Cacchiarelli D. (2014). Perturbation of m6A Writers Reveals Two Distinct Classes of mRNA Methylation at Internal and 5′ Sites. Cell Rep..

[29] Theler D., Dominguez C., Blatter M., Boudet J., Allain F.H.-T. (2014). Solution structure of the YTH domain in complex with N6-methyladenosine RNA: A reader of methylated RNA. Nucleic Acids Res..

[30] Jia G., Fu Y., Zhao X., Dai Q., Zheng G., Yang Y., Yi C., Lindahl T., Pan T., Yang Y.-G. (2011). N6-Methyladenosine in nuclear RNA is a major substrate of the obesity-associated FTO. Nat. Chem. Biol..

[31] Zhang C., Cheng W., Ren X., Wang Z., Liu X., Li G., Han S., Jiang T., Wu A. (2017). Tumor Purity as an Underlying Key Factor in Glioma. Clin. Cancer Res..

[32] Chen Y., Chen Z., Chen R., Fang C., Zhang C., Ji M., Yang X. (2022). Immunotherapy-based combination strategies for treatment of EGFR-TKI-resistant non-small-cell lung cancer. Futur. Oncol..

[33] Michalczyk K., Cymbaluk-Płoska A. (2020). The Role of Zinc and Copper in Gynecological Malignancies. Nutrients.

[34] Que E.L., Domaille D.W., Chang C.J. (2008). Metals in Neurobiology: Probing Their Chemistry and Biology with Molecular Imaging. Chem. Rev..

[35] Shanbhag V.C., Gudekar N., Jasmer K., Papageorgiou C., Singh K., Petris M.J. (2021). Copper metabolism as a unique vulnerability in cancer. Biochim. Biophys. Acta BBA Mol. Cell Res..

[36] Tsvetkov P., Coy S., Petrova B., Dreishpoon M., Verma A., Abdusamad M., Rossen J., Joesch-Cohen L., Humeidi R., Spangler R.D. (2022). Copper induces cell death by targeting lipoylated TCA cycle proteins. Science.

[37] Liao Y., Zhao J., Bulek K., Tang F., Chen X., Cai G., Jia S., Fox P.L., Huang E., Pizarro T.T. (2020). Inflammation mobilizes copper metabolism to promote colon tumorigenesis via an IL-17-STEAP4-XIAP axis. Nat. Commun..

[38] Paredes S.E.Y., Almeida L.Y., Trevisan G.L., Polanco X.B.J., Silveira H.A., Silva E.V., Segato R.A.B., da Silva L.A.B., Chahud F., León J.E. (2020). Immunohistochemical characterization of immune cell infiltration in paediatric and adult Langerhans cell histiocytosis. Scand. J. Immunol..

[39] Chen F., Han B., Meng Y., Han Y., Liu B., Zhang B., Chang Y., Cao P., Fan Y., Tan K. (2021). Ceruloplasmin correlates with immune infiltration and serves as a prognostic biomarker in breast cancer. Aging.

[40] Lin P.M.F., Hsin M.K. (2020). New T1 classification. Gen. Thorac. Cardiovasc. Surg..

[41] Travis W.D., Asamura H., Bankier A.A., Beasley M.B., Detterbeck F., Flieder D.B., Goo J.M., MacMahon H., Naidich D., Nicholson A.G. (2016). The IASLC Lung Cancer Staging Project: Proposals for Coding T Categories for Subsolid Nodules and Assessment of Tumor Size in Part-Solid Tumors in the Forthcoming Eighth Edition of the TNM Classification of Lung Cancer. J. Thorac. Oncol..

[42] Gonzalez-Lopez E., Vrana K.E. (2020). Dopamine beta-hydroxylase and its genetic variants in human health and disease. J. Neurochem..

[43] Bicker J., Alves G., Fortuna A., Soares-Da-Silva P., Falcão A. (2018). In vitro assessment of the interactions of dopamine β-hydroxylase inhibitors with human P-glycoprotein and Breast Cancer Resistance Protein. Eur. J. Pharm. Sci..

[44] Jen P.Y.P., Dixon J.S. (2000). DbetaH-immunoreactive subepithelial nerves in the vas deferens of prostate cancer patients. J. Anat..

[45] Tokumoto M., Lee J.-Y., Fujiwara Y., Uchiyama M., Satoh M. (2013). Inorganic arsenic induces apoptosis through downregulation of Ube2d genes and p53 accumulation in rat proximal tubular cells. J. Toxicol. Sci..

[46] Hou L., Li Y., Wang Y., Xu D., Cui H., Xu X., Cong Y., Yu C. (2018). UBE2D1 RNA Expression Was an Independent Unfavorable Prognostic Indicator in Lung Adenocarcinoma, but Not in Lung Squamous Cell Carcinoma. Dis. Markers.

[47] Somwar R., Erdjument-Bromage H., Larsson E., Shum D., Lockwood W.W., Yang G., Sander C., Ouerfelli O., Tempst P.J., Djaballah H. (2011). Superoxide dismutase 1 (SOD1) is a target for a small molecule identified in a screen for inhibitors of the growth of lung adenocarcinoma cell lines. Proc. Natl. Acad. Sci. USA.

[48] Juarez J.C., Manuia M., Burnett M.E., Betancourt O., Boivin B., Shaw D.E., Tonks N.K., Mazar A.P., Doñate F. (2008). Superoxide dismutase 1 (SOD1) is essential for H_2_O_2_-mediated oxidation and inactivation of phosphatases in growth factor signaling. Proc. Natl. Acad. Sci. USA.

[49] Zhan P., Shen X.-K., Qian Q., Zhu J.-P., Zhang Y., Xie H.-Y., Xu C.-H., Hao K.-K., Hu W., Xia N. (2012). Down-regulation of lysyl oxidase-like 2 (LOXL2) is associated with disease progression in lung adenocarcinomas. Med Oncol..

[50] Mizuno K., Seki N., Mataki H., Matsushita R., Kamikawaji K., Kumamoto T., Takagi K., Goto Y., Nishikawa R., Kato M. (2016). Tumor-suppressive microRNA-29 family inhibits cancer cell migration and invasion directly targeting LOXL2 in lung squamous cell carcinoma. Int. J. Oncol..

[51] Li F., Song Q.-Z., Zhang Y.-F., Wang X.-R., Cao L.-M., Li N., Zhao L.-X., Zhang S.-X., Zhuang X.-F. (2022). Identifying the EMT-related signature to stratify prognosis and evaluate the tumor microenvironment in lung adenocarcinoma. Front. Genet..

