# Supplementary material for: Validation of a Novel Cuproptosis–Related Prognostic Gene Marker and Differential Expression Associated with Lung Adenocarcinoma
Source: Curr Issues Mol Biol. 2023 Oct 22;45(10):8502–18. doi: 10.3390/cimb45100536 (PMC10605745; doi:10.3390/cimb45100536)
Supplement: Supplementary file 1 [file cimb-45-00536-s001.zip › cimb-2656138-supplementary.pdf]

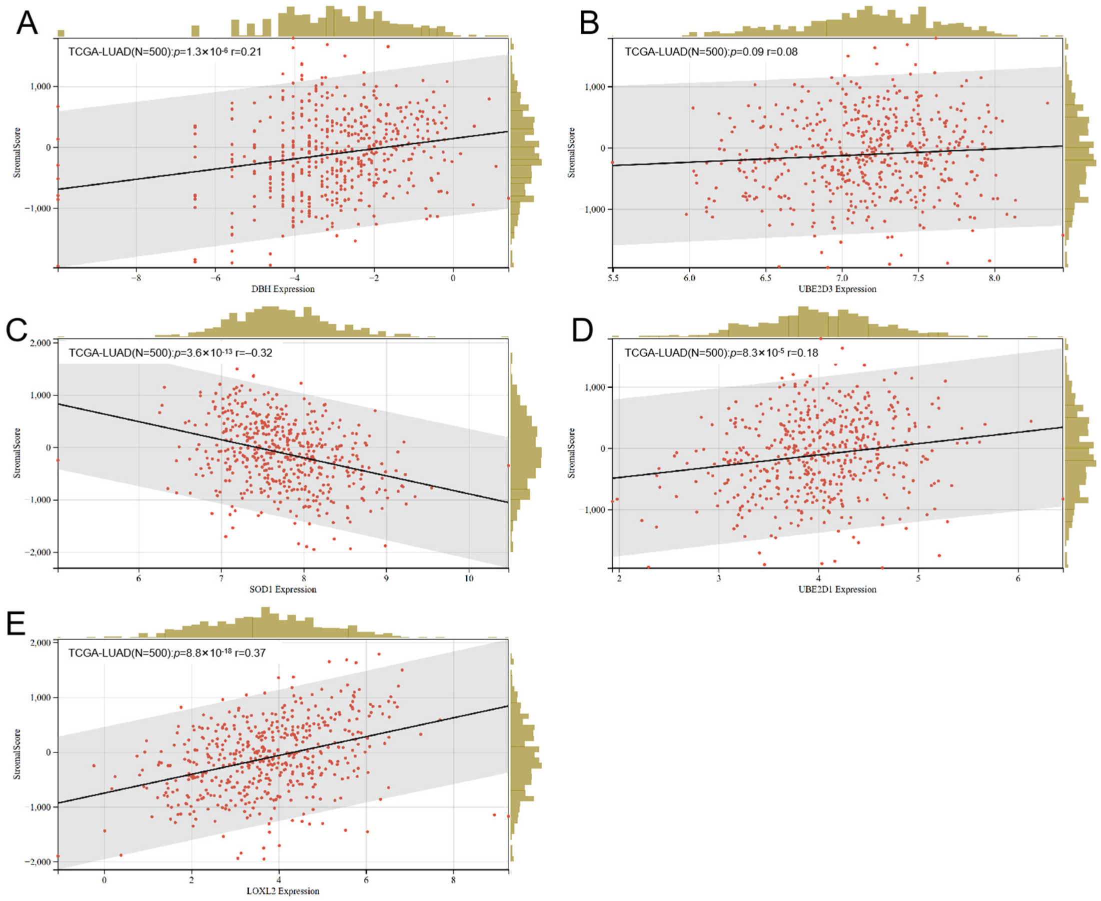

Figure S1. Somatic immunity score between (A) DβH, (B) UBE2D3, (C) SOD1, (D) UBE2D1, and (E) LOXL2 expression.

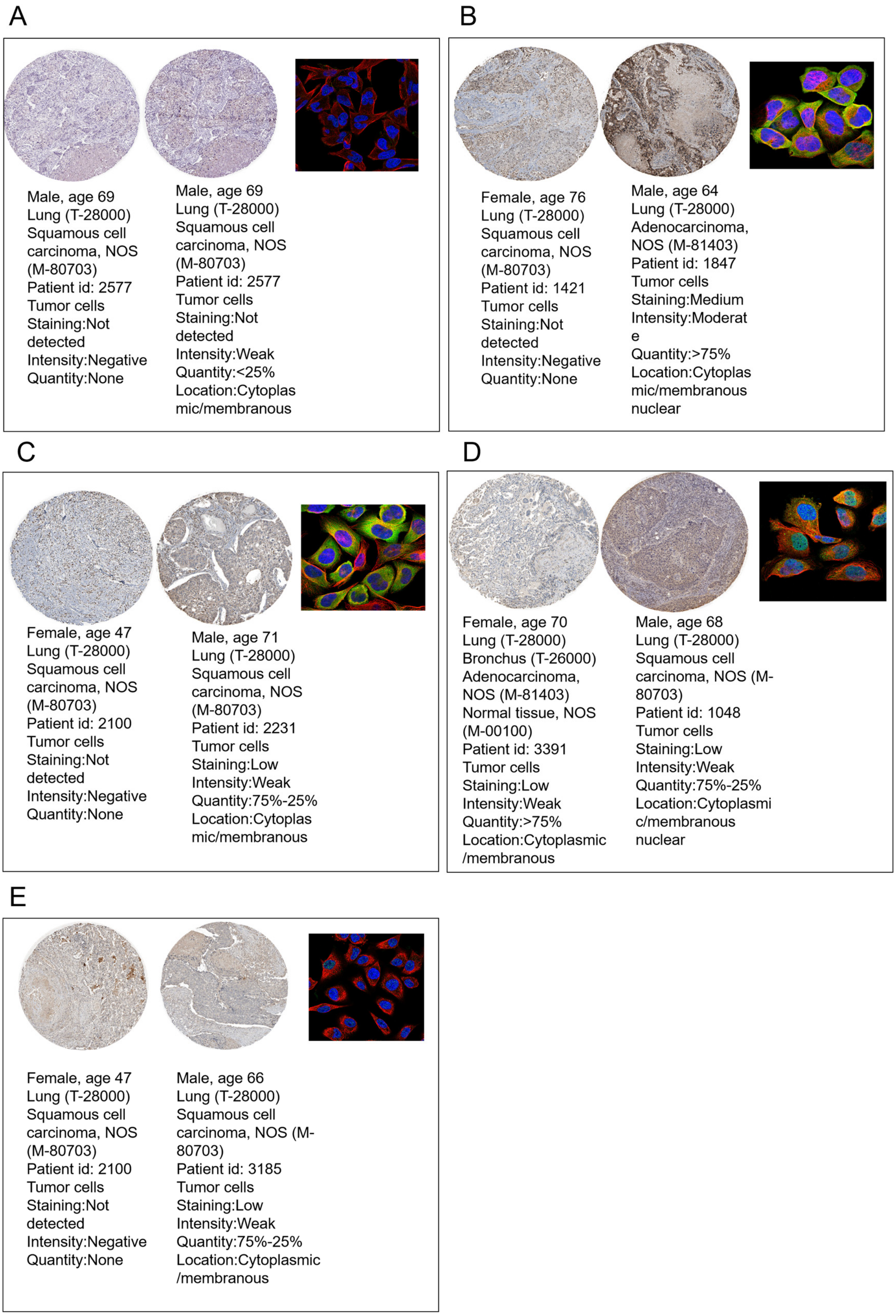

Figure S2. Immunohistochemical staining and immunofluorometric assay, including (A) DβH, (B) UBE2D1, (C) UBE2D3, (D) SOD1, and (E) LOXL2.

Table S1. Results of differential expression of CRGs in GSE10072 and TCGA datasets

Dataset	Gene	log2FC	*p* value	Dataset	Gene	log2FC	*p* value
GSE10072	AOC1	0.58	8.55×10^{-6}	TCGA-LUAD	AOC1	Tumor:2.1±2.92<br>Normal:-2.17±1.94	2.20×10^{-93}
GSE10072	ATOX1	0.15	0.0130	TCGA-LUAD	ATOX1	Tumor:6.85±0.52<br>Normal:6.45±0.95	2.9×10^{-28}
GSE10072	ATP7B	-0.17	9.53×10^{-4}	TCGA-LUAD	ATP7B	Tumor:0.47±1.41<br>Normal:1.06±0.90	5.0×10^{-20}
GSE10072	CCL8	0.40	0.0273	TCGA-LUAD	CCL8	Tumor:1.2±1.80<br>Normal:0.3±2.07	4.2×10^{-11}
GSE10072	CCS	0.12	0.0435	TCGA-LUAD	CCS	-	-
GSE10072	COX11	0.26	0.00702	TCGA-LUAD	COX11	Tumor:4.8±0.52<br>Normal:4.5±0.86	6.2e-7
GSE10072	CP	2.28	2.09×10^{-22}	TCGA-LUAD	CP	Tumor:6.7±2.03<br>Normal:3.1±1.58	1.1×10^{-101}
GSE10072	DβH	-0.11	0.0234	TCGA-LUAD	DβH	Tumor:-3.01±1.67<br>Normal:-0.84±1.23	8.5×10^{-82}
GSE10072	LOXL2	0.82	8.51×10^{-10}	TCGA-LUAD	LOXL2	Tumor:3.72±1.41<br>Normal:2.84±1.12	8.7×10^{-24}
GSE10072	MAP2K2	0.26	0.000176	TCGA-LUAD	MAP2K2	Tumor:6.39±0.49<br>Normal:6.43±0.93	4.1×10^{-3}
GSE10072	PDE3B	-0.59	1.95×10^{-17}	TCGA-LUAD	PDE3B	Tumor:1.13±1.26<br>Normal:2.91±0.88	3.0×10^{-95}
GSE10072	PDK1	0.42	1.18×10^{-10}	TCGA-LUAD	PDK1	Tumor:2.85±0.86<br>Normal:1.90±0.88	1.3×10^{-56}

GSE10072	SCO2	0.21	0.0193	TCGA-LUAD	SCO2	-	-
GSE10072	SLC31A2	-0.76	3.13×10^{-17}	TCGA-LUAD	SLC31A2	Tumor:3.57±0.97 Normal:4.54±1.11	4.0×10^{-50}
GSE10072	SOD1	0.25	9.59×10^{-4}	TCGA-LUAD	SOD1	Tumor:7.74±0.60 Normal:7.31±0.95	6.1×10^{-30}
GSE10072	UBE2D1	0.39	3.76×10^{-7}	TCGA-LUAD	UBE2D1	Tumor:4.00±0.62 Normal:3.80±0.89	1.0×10^{-5}
GSE10072	UBE2D2	-0.10	0.0377	TCGA-LUAD	UBE2D2	Tumor:6.46±0.47 Normal:6.49±0.89	0.01
GSE10072	UBE2D3	0.26	0.000346	TCGA-LUAD	UBE2D3	Tumor:7.17±0.47 Normal:7.55±0.98	2.1×10^{-40}
GSE10072	UBE2D4	-0.06	0.0178	TCGA-LUAD	UBE2D4	Tumor:3.86±0.57 Normal:3.63±0.85	1.0×10^{-5}
GSE10072	ULK2	-0.53	3.73×10^{-13}	TCGA-LUAD	ULK2	Tumor:2.34±0.84 Normal:3.24±0.83	6.1×10^{-67}
GSE10072	VEGFA	0.64	2.77×10^{-6}	TCGA-LUAD	VEGFA	Tumor:6.81±1.09 Normal:7.03±1.12	1.4×10^{-4}

Table S2. The clinical staging of CRGs

Gene	Stage	Compare group (Mean±std)	Control group (Mean±std)	T-test	variance
AOC1	T-stage	T1(2.25±2.69)	T2(2.04±3.06)	0.46	0.1
		T1(2.25±2.69)	T3(3.19±2.78)	**0.04**	
		T1(2.25±2.69)	T4(1.96±2.82)	0.68	
		T2(2.04±3.06)	T3(3.19±2.78)	**0.01**	
		T2(2.04±3.06)	T4(1.96±2.82)	0.91	
		T3(3.19±2.78)	T4(1.96±2.82)	0.13	

	N-Stage	N0(2.29±2.98)	N2(2.00±2.79)	0.43	
		N0(2.29±2.98)	N1(2.10±2.89)	0.58	0.69
		N2(2.00±2.79)	N1(2.10±2.89)	0.82	
	M-stage	M0(2.25±2.85)	M1(2.60±3.26)	0.61	0.61
ATOX1	T-stage	T1(6.84±0.48)	T2(6.85±0.54)	0.88	
		T1(6.84±0.48)	T3(6.83±0.56)	0.95	
		T1(6.84±0.48)	T4(6.91±0.56)	0.60	0.95
		T2(6.85±0.54)	T3(6.83±0.56)	0.89	
		T2(6.85±0.54)	T4(6.91±0.56)	0.64	
		T3(6.83±0.56)	T4(6.91±0.56)	0.62	
	N-Stage	N0(6.81±0.52)	N2(6.93±0.51)	0.09	
		N0(6.81±0.52)	N1(6.94±0.49)	**0.03**	0.05
		N2(6.93±0.51)	N1(6.94±0.49)	0.86	
	M-stage	M0(6.87±0.55)	M1(6.85±0.49)	0.81	0.81
	T-stage	T1(0.38±1.33)	T2(0.51±1.46)	0.36	
		T1(0.38±1.33)	T3(0.53±1.35)	0.52	
		T1(0.38±1.33)	T4(0.46±1.17)	0.81	0.83
		T2(0.51±1.46)	T3(0.53±1.35)	0.93	
		T2(0.51±1.46)	T4(0.46±1.17)	0.86	
ATP7B		T3(0.53±1.35)	T4(0.46±1.17)	0.84	
	N-Stage	N0(0.52±1.43)	N2(0.75±1.19)	0.15	
		N0(0.52±1.43)	N1(0.06±1.37)	**5.1e-3**	3.0e-3
		N2(0.75±1.19)	N1(0.06±1.37)	**6.3e-4**	
	M-stage	M0(0.36±1.41)	M1(0.73±1.74)	0.32	0.32
	T-stage	T1(1.32±1.63)	T2(1.27±1.85)	0.78	
		T1(1.32±1.63)	T3(0.86±1.91)	0.14	
		T1(1.32±1.63)	T4(1.44±2.10)	0.81	0.45
		T2(1.27±1.85)	T3(0.86±1.91)	0.18	
		T2(1.27±1.85)	T4(1.44±2.10)	0.74	
CCL8		T3(0.86±1.91)	T4(1.44±2.10)	0.31	
	N-Stage	N0(1.27±1.79)	N2(1.10±1.84)	0.47	
		N0(1.27±1.79)	N1(1.29±1.79)	0.93	0.74
		N2(1.10±1.84)	N1(1.29±1.79)	0.50	
	M-stage	M0(1.27±1.84)	M1(1.67±1.97)	0.33	0.33
	T-stage	T1(5.28±0.61)	T2(5.18±0.66)	0.12	
		T1(5.28±0.61)	T3(5.10±0.54)	0.05	
		T1(5.28±0.61)	T4(4.90±0.57)	**0.01**	0.04
		T2(5.18±0.66)	T3(5.10±0.54)	0.33	
		T2(5.18±0.66)	T4(4.90±0.57)	0.06	
CCS		T3(5.10±0.54)	T4(4.90±0.57)	0.21	
	N-Stage	N0(5.21±0.65)	N2(5.16±0.64)	0.54	
		N0(5.21±0.65)	N1(5.20±0.59)	0.90	0.82
		N2(5.16±0.64)	N1(5.20±0.59)	0.66	
	M-stage	M0(5.16±0.61)	M1(5.09±0.62)	0.55	0.55

COX11	T-stage	T1(4.83±0.51)	T2(4.82±0.53)	0.92	0.62
		T1(4.83±0.51)	T3(4.72±0.57)	0.24	
		T1(4.83±0.51)	T4(4.86±0.54)	0.83	
		T2(4.82±0.53)	T3(4.72±0.57)	0.25	
		T2(4.82±0.53)	T4(4.86±0.54)	0.80	
		T3(4.72±0.57)	T4(4.86±0.54)	0.38	
	N-Stage	N0(4.83±0.53)	N2(4.85±0.49)	0.75	0.73
		N0(4.83±0.53)	N1(4.79±0.54)	0.51	
		N2(4.85±0.49)	N1(4.79±0.54)	0.44	
	M-stage	M0(4.84±0.51)	M1(4.88±0.53)	0.72	0.72
CP	T-stage	T1(6.80±1.94)	T2(6.73±1.98)	0.72	0.42
		T1(6.80±1.94)	T3(6.26±2.16)	0.13	
		T1(6.80±1.94)	T4(6.53±3.08)	0.72	
		T2(6.73±1.98)	T3(6.26±2.16)	0.17	
		T2(6.73±1.98)	T4(6.53±3.08)	0.79	
		T3(6.26±2.16)	T4(6.53±3.08)	0.74	
	N-Stage	N0(6.62±1.98)	N2(6.58±2.27)	0.89	0.11
		N0(6.62±1.98)	N1(7.09±1.99)	**0.04**	
		N2(6.58±2.27)	N1(7.09±1.99)	0.13	
	M-stage	M0(6.69±2.10)	M1(6.59±1.97)	0.81	0.81
DβH	T-stage	T1(-2.42±1.42)	T2(-3.29±1.72)	**1.8e-8**	2.9e-7
		T1(-2.42±1.42)	T3(-3.29±1.63)	**1.5e-3**	
		T1(-2.42±1.42)	T4(-3.66±1.95)	**0.02**	
		T2(-3.29±1.72)	T3(-3.29±1.63)	0.99	
		T2(-3.29±1.72)	T4(-3.66±1.95)	0.44	
		T3(-3.29±1.63)	T4(-3.66±1.95)	0.48	
	N-Stage	N0(-2.78±1.64)	N2(-3.64±1.69)	**1.2e-4**	3.9e-5
		N0(-2.78±1.64)	N1(-3.33±1.67)	**5.0e-3**	
		N2(-3.64±1.69)	N1(-3.33±1.67)	0.23	
	M-stage	M0(-3.09±1.63)	M1(-3.24±2.27)	0.75	0.75
LOXL2	T-stage	T1(3.46±1.28)	T2(3.76±1.46)	**0.02**	5.8e-4
		T1(3.46±1.28)	T3(3.96±1.32)	**0.03**	
		T1(3.46±1.28)	T4(4.78±1.55)	**2.4e-3**	
		T2(3.76±1.46)	T3(3.96±1.32)	0.37	
		T2(3.76±1.46)	T4(4.78±1.55)	**0.01**	
		T3(3.96±1.32)	T4(4.78±1.55)	0.06	
	N-Stage	N0(3.58±1.38)	N2(4.04±1.43)	**0.01**	4.9e-3
		N0(3.58±1.38)	N1(4.00±1.44)	**0.01**	
		N2(4.04±1.43)	N1(4.00±1.44)	0.86	
	M-stage	M0(3.73±1.43)	M1(3.91±1.58)	0.59	0.59
MAP2K2	T-stage	T1(6.39±0.45)	T2(6.38±0.52)	0.84	0.51
		T1(6.39±0.45)	T3(6.49±0.48)	0.22	
		T1(6.39±0.45)	T4(6.32±0.44)	0.51	
		T2(6.38±0.52)	T3(6.49±0.48)	0.18	

		T2(6.38±0.52)	T4(6.32±0.44)	0.56	
		T3(6.49±0.48)	T4(6.32±0.44)	0.18	
	N-Stage	N0(6.38±0.49)	N2(6.46±0.45)	0.18	
		N0(6.38±0.49)	N1(6.43±0.48)	0.32	0.34
		N2(6.46±0.45)	N1(6.43±0.48)	0.74	
	M-stage	M0(6.39±0.50)	M1(6.23±0.41)	0.07	0.07
PDE3B	T-stage	T1(1.26±1.14)	T2(1.11±1.31)	0.22	
		T1(1.26±1.14)	T3(0.99±1.34)	0.22	
		T1(1.26±1.14)	T4(0.61±1.37)	0.07	0.15
		T2(1.11±1.31)	T3(0.99±1.34)	0.58	
		T2(1.11±1.31)	T4(0.61±1.37)	0.15	
		T3(0.99±1.34)	T4(0.61±1.37)	0.33	
	N-Stage	N0(1.28±1.27)	N2(0.97±1.25)	0.05	
		N0(1.28±1.27)	N1(0.70±1.15)	**3.6e-5**	1.8e-4
		N2(0.97±1.25)	N1(0.70±1.15)	0.16	
	M-stage	M0(1.14±1.28)	M1(1.05±1.43)	0.78	0.78
PDK1	T-stage	T1(2.80±0.85)	T2(2.91±0.85)	0.19	
		T1(2.80±0.85)	T3(2.68±0.83)	0.38	
		T1(2.80±0.85)	T4(2.83±0.97)	0.91	0.28
		T2(2.91±0.85)	T3(2.68±0.83)	0.08	
		T2(2.91±0.85)	T4(2.83±0.97)	0.73	
		T3(2.68±0.83)	T4(2.83±0.97)	0.57	
	N-Stage	N0(2.84±0.87)	N2(2.85±0.88)	0.92	
		N0(2.84±0.87)	N1(2.87±0.81)	0.73	0.94
		N2(2.85±0.88)	N1(2.87±0.81)	0.86	
	M-stage	M0(2.84±0.86)	M1(2.99±0.94)	0.44	0.44
SCO2	T-stage	T1(5.20±0.66)	T2(5.23±0.69)	0.71	
		T1(5.20±0.66)	T3(5.29±0.64)	0.42	
		T1(5.20±0.66)	T4(4.99±0.70)	0.23	0.44
		T2(5.23±0.69)	T3(5.29±0.64)	0.55	
		T2(5.23±0.69)	T4(4.99±0.70)	0.18	
		T3(5.29±0.64)	T4(4.99±0.70)	0.12	
	N-Stage	N0(5.19±0.68)	N2(5.29±0.65)	0.23	
		N0(5.19±0.68)	N1(5.29±0.65)	0.18	0.28
		N2(5.29±0.65)	N1(5.29±0.65)	0.99	
	M-stage	M0(5.24±0.65)	M1(5.33±0.55)	0.44	0.44
SLC31A2	T-stage	T1(3.67±0.85)	T2(3.52±1.01)	0.10	
		T1(3.67±0.85)	T3(3.55±0.93)	0.44	
		T1(3.67±0.85)	T4(3.17±1.24)	0.11	0.13
		T2(3.52±1.01)	T3(3.55±0.93)	0.84	
		T2(3.52±1.01)	T4(3.17±1.24)	0.25	
		T3(3.55±0.93)	T4(3.17±1.24)	0.24	
	N-Stage	N0(3.55±0.97)	N2(3.55±0.88)	0.97	0.91
		N0(3.55±0.97)	N1(3.60±1.04)	0.70	

		N2(3.55±0.88)	N1(3.60±1.04)	0.73	
	M-stage	M0(3.54±0.97)	M1(3.29±1.25)	0.34	0.34
SOD1	T-stage	T1(7.78±0.59)	T2(7.72±0.61)	0.29	0.48
		T1(7.78±0.59)	T3(7.72±0.57)	0.51	
		T1(7.78±0.59)	T4(7.90±0.71)	0.48	
		T2(7.72±0.61)	T3(7.72±0.57)	0.99	
		T2(7.72±0.61)	T4(7.90±0.71)	0.29	
		T3(7.72±0.57)	T4(7.90±0.71)	0.33	
	N-Stage	N0(7.73±0.59)	N2(7.74±0.56)	0.89	0.46
		N0(7.73±0.59)	N1(7.82±0.66)	0.25	
		N2(7.74±0.56)	N1(7.82±0.66)	0.42	
	M-stage	M0(7.73±0.60)	M1(8.00±0.59)	**0.04**	0.04
UBE2D1	T-stage	T1(3.91±0.61)	T2(4.04±0.64)	**0.03**	0.06
		T1(3.91±0.61)	T3(4.01±0.57)	0.27	
		T1(3.91±0.61)	T4(4.23±0.58)	**0.03**	
		T2(4.04±0.64)	T3(4.01±0.57)	0.81	
		T2(4.04±0.64)	T4(4.23±0.58)	0.18	
		T3(4.01±0.57)	T4(4.23±0.58)	0.18	
	N-Stage	N0(3.96±0.61)	N2(4.13±0.69)	0.05	0.06
		N0(3.96±0.61)	N1(4.06±0.59)	0.16	
		N2(4.13±0.69)	N1(4.06±0.59)	0.45	
	M-stage	M0(4.02±0.58)	M1(4.05±0.71)	0.85	0.85
UBE2D2	T-stage	T1(6.44±0.40)	T2(6.47±0.50)	0.57	0.16
		T1(6.44±0.40)	T3(6.38±0.47)	0.41	
		T1(6.44±0.40)	T4(6.66±0.49)	0.08	
		T2(6.47±0.50)	T3(6.38±0.47)	0.25	
		T2(6.47±0.50)	T4(6.66±0.49)	0.12	
		T3(6.38±0.47)	T4(6.66±0.49)	**0.04**	
	N-Stage	N0(6.45±0.49)	N2(6.50±0.42)	0.31	0.62
		N0(6.45±0.49)	N1(6.47±0.45)	0.69	
		N2(6.50±0.42)	N1(6.47±0.45)	0.59	
	M-stage	M0(6.48±0.46)	M1(6.56±0.38)	0.30	0.30
UBE2D3	T-stage	T1(7.12±0.43)	T2(7.18±0.49)	0.15	0.10
		T1(7.12±0.43)	T3(7.18±0.42)	0.34	
		T1(7.12±0.43)	T4(7.38±0.51)	0.05	
		T2(7.18±0.49)	T3(7.18±0.42)	0.97	
		T2(7.18±0.49)	T4(7.38±0.51)	0.12	
		T3(7.18±0.42)	T4(7.38±0.51)	0.15	
	N-Stage	N0(7.14±0.48)	N2(7.21±0.42)	0.19	0.08
		N0(7.14±0.48)	N1(7.25±0.43)	**0.03**	
		N2(7.21±0.42)	N1(7.25±0.43)	0.55	
	M-stage	M0(7.19±0.47)	M1(7.35±0.38)	0.05	0.05
UBE2D4	T-stage	T1(3.87±0.53)	T2(3.88±0.60)	0.77	0.55
		T1(3.87±0.53)	T3(3.75±0.54)	0.20	

		T1(3.87±0.53)	T4(3.86±0.68)	0.98	
		T2(3.88±0.60)	T3(3.75±0.54)	0.13	
		T2(3.88±0.60)	T4(3.86±0.68)	0.91	
		T3(3.75±0.54)	T4(3.86±0.68)	0.54	
	N-Stage	N0(3.81±0.54)	N2(3.99±0.60)	**0.02**	
		N0(3.81±0.54)	N1(3.92±0.64)	0.15	0.03
		N2(3.99±0.60)	N1(3.92±0.64)	0.46	
	M-stage	M0(3.88±0.61)	M1(3.87±0.47)	0.92	0.92
ULK2	T-stage	T1(2.45±0.77)	T2(2.30±0.81)	0.05	
		T1(2.45±0.77)	T3(2.10±1.01)	**0.03**	
		T1(2.45±0.77)	T4(2.26±1.21)	0.53	0.06
		T2(2.30±0.81)	T3(2.10±1.01)	0.21	
		T2(2.30±0.81)	T4(2.26±1.21)	0.89	
		T3(2.10±1.01)	T4(2.26±1.21)	0.62	
	N-Stage	N0(2.34±0.83)	N2(2.37±0.85)	0.81	
		N0(2.34±0.83)	N1(2.22±0.86)	0.21	0.39
		N2(2.37±0.85)	N1(2.22±0.86)	0.26	
	M-stage	M0(2.29±0.87)	M1(2.44±0.88)	0.41	0.41
VEGFA	T-stage	T1(6.77±1.07)	T2(6.81±1.14)	0.66	
		T1(6.77±1.07)	T3(6.78±0.97)	0.95	
		T1(6.77±1.07)	T4(7.21±0.95)	0.08	0.44
		T2(6.81±1.14)	T3(6.78±0.97)	0.81	
		T2(6.81±1.14)	T4(7.21±0.95)	0.11	
		T3(6.78±0.97)	T4(7.21±0.95)	0.11	
	N-Stage	N0(6.77±1.09)	N2(6.89±1.05)	0.36	
		N0(6.77±1.09)	N1(6.91±1.11)	0.26	0.42
		N2(6.89±1.05)	N1(6.91±1.11)	0.90	
	M-stage	M0(6.79±1.12)	M1(6.96±0.99)	0.43	0.43
